# Minimally Invasive Subcortical Parafascicular Transsulcal Access for Clot Evacuation (Mi SPACE) for Intracerebral Hemorrhage

**DOI:** 10.1155/2014/102307

**Published:** 2014-08-06

**Authors:** Benjamin Ritsma, Amin Kassam, Dariush Dowlatshahi, Thanh Nguyen, Grant Stotts

**Affiliations:** ^1^Division of Physical Medicine & Rehabilitation, University of Ottawa, The Ottawa Hospital, Ottawa, ON, Canada K1H 8M2; ^2^Division of Neurosurgery, University of Ottawa, The Ottawa Hospital, Ottawa, ON, Canada K1H 8L6; ^3^Division of Neurology, University of Ottawa, The Ottawa Hospital, Civic Campus, 1053 Carling Avenue, Room C2184, Ottawa, ON, Canada K1Y 4E9; ^4^Division of Neuroradiology, University of Ottawa, The Ottawa Hospital, Ottawa, ON, Canada K1H 8L6

## Abstract

*Background*. Spontaneous intracerebral hemorrhage (ICH) is common and causes significant mortality and morbidity. To date, optimal medical and surgical intervention remains uncertain. A lack of definitive benefit for operative management may be attributable to adverse surgical effect, collateral tissue injury. This is particularly relevant for ICH in dominant, eloquent cortex. Minimally invasive surgery (MIS) offers the potential advantage of reduced collateral damage. MIS utilizing a parafascicular approach has demonstrated such benefit for intracranial tumor resection. *Methods*. We present a case of dominant hemisphere spontaneous ICH evacuated via the minimally invasive subcortical parafascicular transsulcal access clot evacuation (Mi SPACE) model. We use this report to introduce Mi SPACE and to examine the application of this novel MIS paradigm. *Case Presentation*. The featured patient presented with a left temporal ICH and severe global aphasia. The hematoma was evacuated via the Mi SPACE approach. Postoperative reassessments showed significant improvement. At two months, bedside language testing was normal. MRI tractography confirmed limited collateral injury. *Conclusions*. This case illustrates successful application of the Mi SPACE model to ICH in dominant, eloquent cortex and subcortical regions. MRI tractography illustrates collateral tissue preservation. Safety and feasibility studies are required to further assess this promising new therapeutic paradigm.

## 1. Introduction

Spontaneous intracerebral hemorrhage (ICH) affects approximately 2 million people worldwide each year [1]. Overall ICH mortality rate exceeds 40% and only 25% of survivors achieve full independence at 6 months [2]. Despite significant efforts, optimal medical and surgical ICH care remains uncertain [3, 4].

A lack of definitive benefit for operative management may be attributable to adverse surgical effect, collateral tissue injury [5]. This is particularly relevant for ICH in dominant, eloquent cortex and deep regions. Minimally invasive surgery (MIS) offers potential advantages over conventional craniotomy and several groups have examined such techniques [3, 4]. Results have been conflicting and improved functional outcome has not been consistently demonstrated [3, 4]. Minimally invasive subcortical parafascicular transsulcal access for clot evacuation (Mi SPACE) is a novel paradigm designed to address key challenges in intracranial MIS. Earlier iterations of this approach have demonstrated safety and efficacy in treating diverse subcortical lesions [6].

We present the initial case of ICH evacuated via the Mi SPACE model. We use this report to introduce Mi SPACE and to examine its application to ICH in dominant, eloquent cortical and subcortical regions.

## 2. Case

A 64-year-old right-hand dominant male presented to the emergency department with a one-day history of progressive left-sided headache and aphasia. He was normotensive, awake, and alert but unable to express orientation. He was nonfluent and unable to follow 1-step verbal commands, repeat, name, read, or write a sentence. CT head imaging revealed a large left temporal hematoma. CT angiography denoted the absence of an identifiable vascular etiology. The patient was admitted for monitoring and blood pressure regulation.

Past medical history was significant for an occult bleeding diathesis, having presented over the past decade with two other ICH episodes. These events were conservatively managed and resulted in mild residual left-sided motor and sensory deficits.

**Figure 1:**
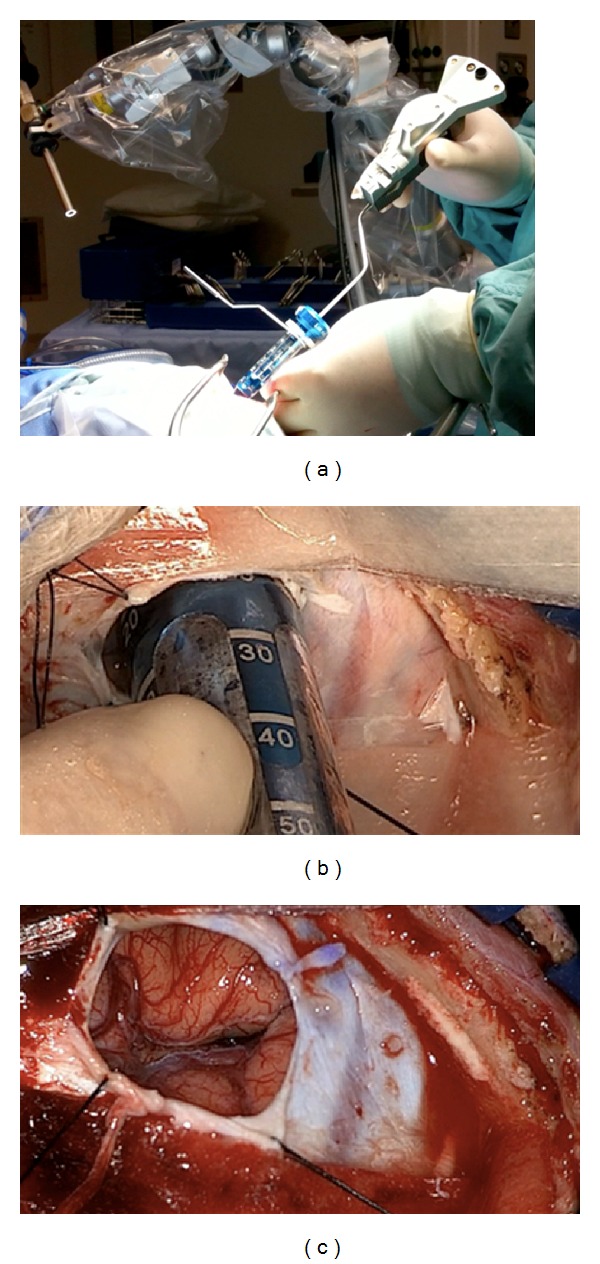
Intraoperative images depicting (a) the Mi SPACE computer assisted navigation system and the radial access transsulcal corridor system, BrainPath (BP) (NICO corp., Indianapolis, Indiana), (b) BrainPath (BP), and (c) the small craniotomy site and dime-sized dural opening created along the predefined sulcus for an entry point. A narrow dural opening allows for a tight seal against the edges of the BP.

On postadmission day three the patient had several episodes of emesis and his headache worsened. A repeat CT head showed a hematomal expansion with mass effect and impending uncal herniation (see Figure 1 in Supplementary Materials available online at http://dx.doi.org/10.1155/2014/102307). Given the risk of further deterioration, prompt surgical intervention was deemed necessary. As the hematoma centred upon highly eloquent cortex with subcortical extension, the Mi SPACE approach was favoured. Starting with neuronavigation, Mi SPACE (Figures 1(a)–1(c)) was employed as outlined below. Following intervention, target systolic blood pressure (SBP) was less than 140 mm Hg.

On the same day postoperative assessment established significant improvement. The patient expressed orientation, followed one-step verbal commands, and used full sentences. Testing on the NIH Stroke Scale (NIHSS) was as follows: repetition 4/6, naming 1/6, and reading intact. CT imaging showed drainage of the hematoma, with improved mass effect (Figure 2 in Supplementary Materials).

In the following evening there was an episode of hypertension, peak SBP 173 mm Hg, requiring administration of intravenous (IV) antihypertensive agents. The patient subsequently developed recurrent emesis and worsening aphasia, approaching admission status. Repeat CT imaging revealed reaccumulation of the hematoma with added mass effect (Figure 3 in Supplementary Materials). As such, he returned for a second evacuation using Mi SPACE. Given this patient's occult bleeding diathesis and history of recurrent ICH, this course of perioperative management included antifibrinolytic agent tranexamic acid (Cyklokapron), 1 g IV loading dose followed by 300 mg IV every eight hours for forty-eight hours, in conjunction with tight blood pressure control (parameters of SBP less than 130 mm Hg for forty-eight hours, then SBP less than 140 mm Hg).

**Figure 2:**
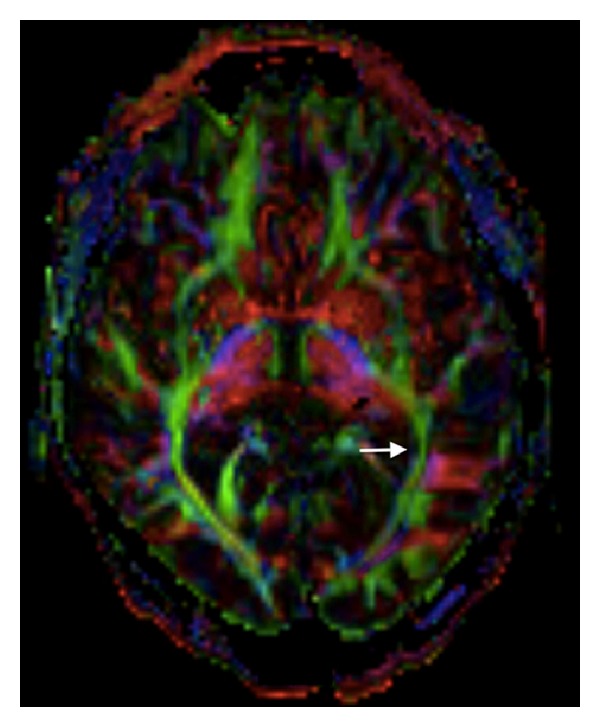
Color coded fractional anisotropy (FA) maps obtained from the diffusion-tensor imaging (DTI) show collateral tissue preservation, with only small areas of decrease in FA in the left inferior longitudinal fasciculus (long arrow).

Again, postsurgical examination demonstrated significant improvement and on same-day CT imaging the hemorrhage was no longer visualized (Figure 4 in Supplementary Materials). On postoperative day one he was awake and alert and expressed full orientation. He followed one- and two-step commands and communicated in full sentences. Naming and repetition were intact on NIHSS. At a two-month follow-up, NIHSS language testing was normal, and he had returned to his baseline level of function. At nine months, MRI with tractography demonstrated a small surgical tract extending from the left temporal cortex to the atrium of the lateral ventricle (Figure 5 in Supplementary Materials). Fractional anisotropy (FA) maps revealed minimal areas of decrease in FA in the left inferior longitudinal fasciculus and left temporal subcortical white matter (Figure 2).

## 3. Discussion

Spontaneous ICH is relatively common and it causes significant mortality and morbidity [1, 2]. Despite decades of quality research, optimal management strategies remain uncertain [3]. Neurosurgical intervention remains controversial and quite variable in practice, particularly for supratentorial hematomas [3, 4].

Though inconsistent, there is data supporting potential benefits of surgical intervention in ICH [4]. As such, identifying patient subgroups more likely to show improved postsurgical outcome is the subject of ongoing investigation [7]. In a survey of British neurosurgeons, ICH dominance and depth affected clinical decision making over and above all other features [8]. Traditionally, a nonoperative approach has been favoured for dominant hemisphere and deep hematomas [3, 9]. Hesitation for surgery in this context is attributable to the fact that operative removal by standard craniotomy almost always requires creating access by transecting through uninjured brain [3]. Indeed, adverse surgical effect has been postulated as a potential explanation for the inability to prove benefit with hematoma evacuation [5].

In an attempt to limit tissue damage and reduce surgical morbidity, MIS techniques have been analyzed [3, 4]. Several groups have reported benefits and neurosurgeons have expressed optimism for such implementation [4, 9]. Nevertheless, improved outcomes have not been consistently demonstrated [3, 4]. A key challenge has been the ability to safely access the hematoma, particularly if in the subcortical space, through a MIS corridor that allows for adequate visualization and bimanual technique to remove early fibrotic clots, without the use of thrombolytics. Moreover, previous endoscopic designs have facilitated a less complete removal of hematoma volume compared to standard craniotomy [10].

Mi SPACE represents an integration of 5 core technologies ((1) Mapping, (2) Navigation, (3) Access, (4) Optics, and (5) Resection) into a single platform to address such challenges. Preoperative mapping via MRI tractography allows the calculation of a surgical trajectory, along the long axis of the most eloquent fibres, to minimize shear forces and fascicle injury. Intraoperative neuronavigation facilitates a transsulcal insertion of a 13.5 mm port (BrainPath, NICO corp., Indianapolis, Indiana), also specifically designed to minimize strain forces, along the preplanned trajectory. This creates a parafascicular corridor that enables (i) use of a novel telescopic optics system, Video Telescopic Assisted Microscopy (VTOM) (Storz corp., Culver City, CA), to optimize visualization, (ii) bimanual dissection technique, and (iii) use of specifically designed nonthermal automated MIS instrumentation (Myriad, NICO corp., Indianapolis, Indiana) without the need for thrombolytics.

This case illustrates the successful initial application of the Mi SPACE approach to ICH in dominant, eloquent cortical and subcortical regions, in a patient with an occult bleeding diathesis. There was clear survival benefit as well as a dramatic early and lasting functional response. MRI tractography demonstrated limited impact on the eloquent fibre tracts, including the arcuate fasciculus, despite the initial hemorrhage, ICH recurrence, and two Mi SPACE evacuations.

Intraoperatively, clot over the arcuate fasciculus was removed and we postulate that removal of this irritant effect was largely responsible for the improved outcome. Although this case's hematoma had a lobar focus, with superficial elements, there was also considerable subcortical extension. These deeper components were also targeted in the evacuation. Earlier iterations of the Mi SPACE model have shown safety and efficacy in managing various subcortical lesions [6]. Thus, in addition to demonstrating potential value in treating dominant, eloquent cortical bleeds, this case may serve as a proof of concept for application to ICH with a subcortical focus.

It has been suggested that a greater volume of hematoma removal is associated with better outcome [10]. The potential for Mi SPACE to offer improved visualization and bimanual technique may facilitate a more complete evacuation than previous MIS designs. In turn, this may further reduce ICH mechanical and toxic effects on adjacent tissue, including white matter tracts, to a degree sufficient enough to derive improved clinical outcomes.

Mi SPACE allowed for adequate intraoperative hemostasis in both interventions, despite the patient's occult bleeding diathesis, and there was excellent postoperative functional recovery. Furthermore, after clot reaccumulation, presumed to be from a combination of postoperative hypertension and the diathesis, repeat surgery within twenty-four hours of initial intervention yielded even better clinical and radiological outcomes. Typically, this is the period in which postoperative edema from conventional surgery becomes of greatest concern. It should also be noted that, in both instances of intervention, the patient underwent early surgery, within hours of clinical deterioration, which may offer benefit in some ICH subgroups [3].

Perioperative management following the second surgery included antifibrinolytic treatment of the bleeding diathesis and more aggressive blood pressure control, after which there was no recurrent bleeding. Additional experience with Mi SPACE could further assess these aspects of ICH medical management.

## 4. Conclusion

This case illustrates the successful initial application of the Mi SPACE model to ICH, including those in dominant, eloquent cortex and subcortical regions. There was clear survival benefit and dramatic functional response. MRI tractography demonstrates collateral fascicle preservation. Safety and feasibility studies are required to further assess this promising new surgical paradigm in ICH care.

## Supplementary Material

Mindful of manuscript length, the authors have included several additional figures as Supplementary Materials. These images contribute to further case description.
