# Supplementary material for: Minimally Invasive Subcortical Parafascicular Transsulcal Access for Clot Evacuation (Mi SPACE) for Intracerebral Hemorrhage
Source: Case Rep Neurol Med. 2014 Aug 6;2014:102307. doi: 10.1155/2014/102307 (PMC4140146; doi:10.1155/2014/102307)
Supplement: Supplementary file 1 — Mindful of manuscript length, the authors have included several additional figures as Supplementary Materials. These images contribute to further case description. [file 102307.f1.docx]

**Figure 1**

Axial CT image demonstrates an enlarging hematoma in left temporal lobe with significant mass effect.

**
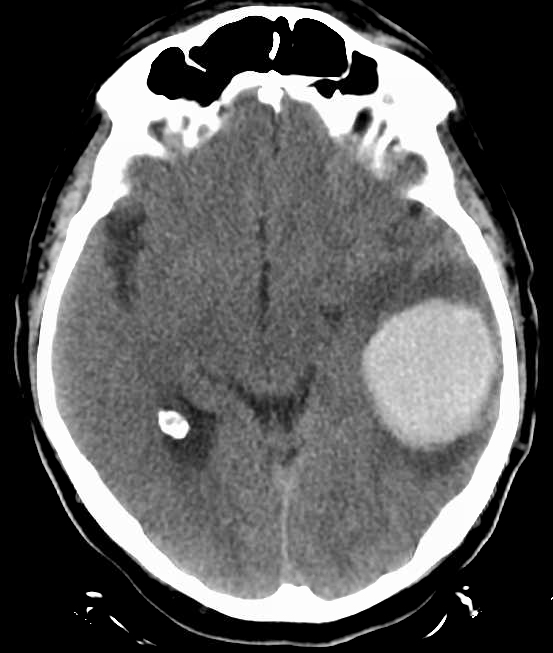
**

**Figure 2**

Post-operative CT image reveals drainage of hematoma and improved mass effect.

During this initial operation Mi SPACE was used to remove the majority of clot. A decision was made to leave the depth along the arcuate fasciculus once mass effect was resolved.

**
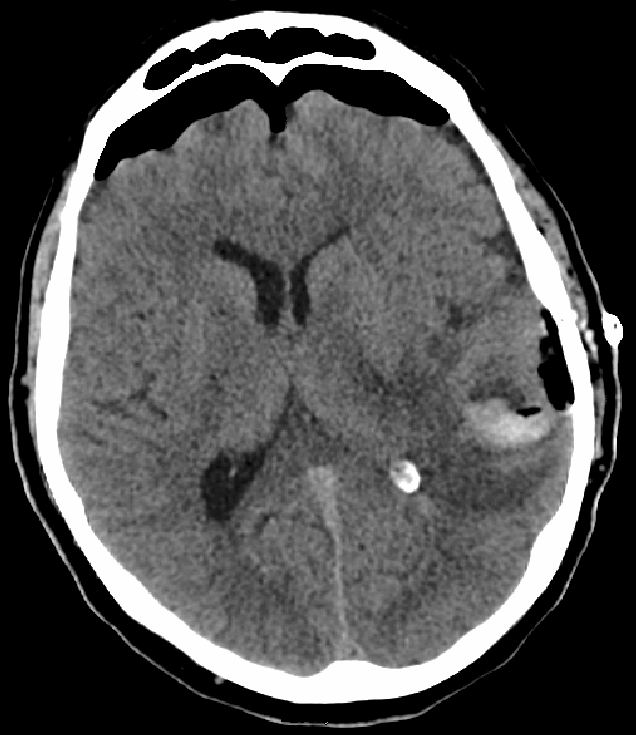
**

**Figure 3**

Repeat CT shows reaccumulation of the left temporal hematoma.

**
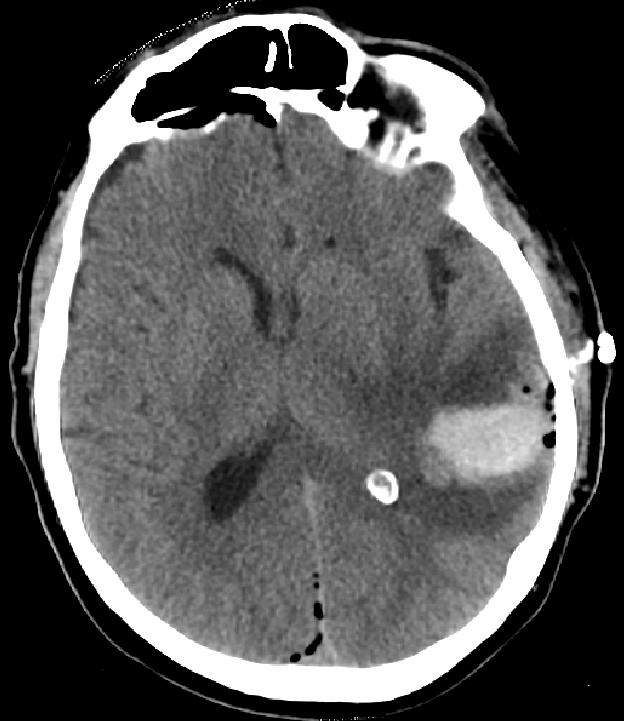
**

**Figure 4**

Post-operative CT reveals hematoma evacuation with the hemorrhage no longer visualized.

**
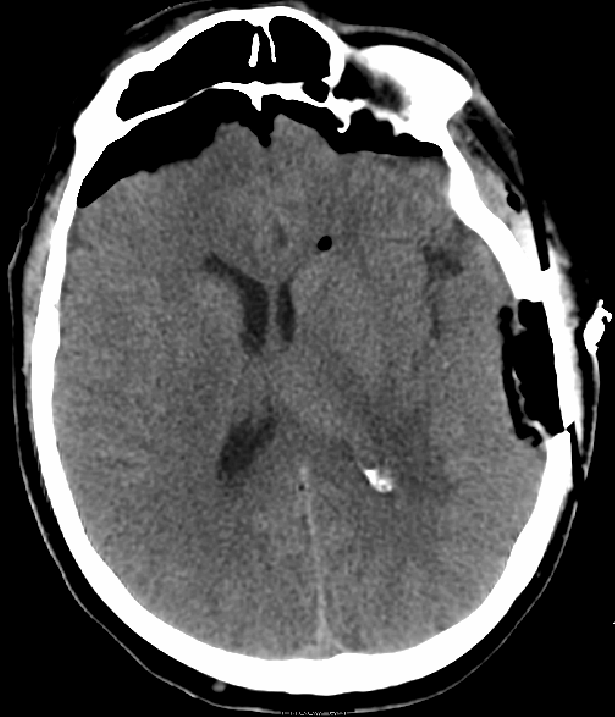
**

**Figure 5**

Axial T2-weighted image demonstrates a small surgical tract extending from the left temporal cortex to the atrium of the lateral ventricle.

**
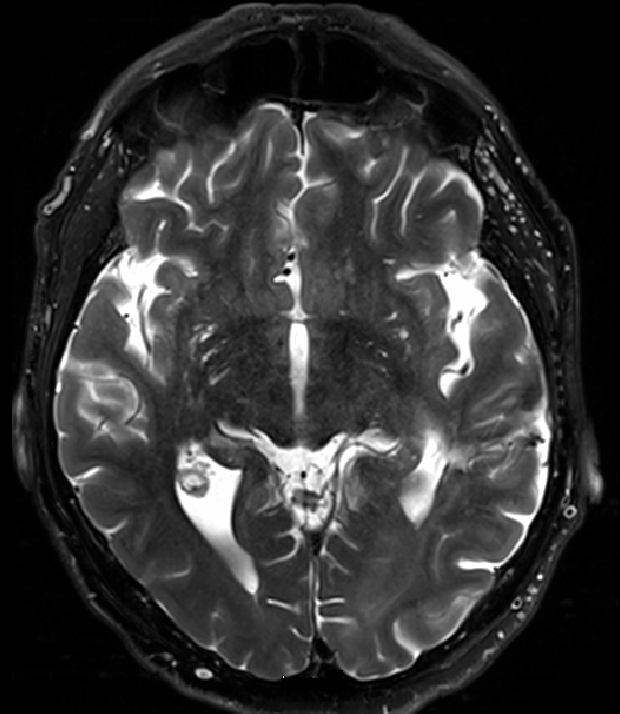
**
